# Utilization of a New Intracranial Support Catheter as an Intermediate Aspiration Catheter in the Treatment of Acute Ischemic Stroke: Technical Report on Initial Experience

**DOI:** 10.7759/cureus.617

**Published:** 2016-05-21

**Authors:** J. Diego Lozano, Francesco Massari, Mary C Howk, Katyucia de Macedo Rodrigues, Christopher Brooks, Mary Perras, David E Rex, Ajay K Wakhloo, Anna Luisa Kühn, Ajit S Puri

**Affiliations:** 1 Division of Neuroimaging and Intervention, Department of Radiology, University of Massachusetts

**Keywords:** acute ischemic stroke, large vessel occlusion, mechanical thrombectomy, catheter, endovascular treatment, device, revascularization, intracranial support catheter, intermediate catheter, triaxial technique

## Abstract

The endovascular management of acute ischemic stroke (AIS) due to emergency large vessel occlusion (ELVO) has become the standard of care after the recent publication of landmark randomized, controlled trials. Mechanical thrombectomy, in addition to intravenous thrombolysis (within 4.5 hours when eligible), is now part of the algorithm of the standard of care when treating AIS in patients with ELVO in the anterior circulation up to six hours after symptom onset. A newly introduced device, the Arc™ intracranial support catheter (Medtronic, Irvine, USA), is specifically designed for the introduction of neurointerventional devices into the cerebral vasculature and facilitates the delivery of microcatheters into smaller, more distal intracranial vessels. This technical report describes the use of the Arc™ intracranial support catheter in the setting of AIS.

## Introduction

Mechanical thrombectomy using stent-retrievers and/or a large-bore aspiration catheter is now considered part of the standard-of-care algorithm for carefully selected patients presenting with acute ischemic stroke (AIS) secondary to emergency large vessel occlusion (ELVO) (Class IIa; Level of Evidence C), as indicated by the recent 2015 American Heart Association/American Stroke Association Focused Update of the 2013 Guidelines for the Early Management of Patients With Acute Ischemic Stroke Regarding Endovascular Treatment [1]. Several landmark randomized, controlled clinical studies were recently published regarding the effectiveness of these devices not only for early recanalization of an occluded intracranial artery in the anterior circulation but, most importantly, for significant improvement in time to reperfusion, which ultimately results in marked reduction of morbidity and mortality related to AIS secondary to ELVO [2-6]. Different techniques to achieve recanalization of an ELVO in the anterior circulation have been proposed and are in current use in clinical practice worldwide. These techniques primarily include: (1) Mechanical thrombectomy through a balloon guide catheter (BGC) positioned at the cervical internal carotid artery (ICA); (2) mechanical thrombectomy in conjunction with thromboaspiration (Solumbra technique); (3) mechanical thrombectomy through a guide catheter with the tip placed in the cervical ICA; and (4) a direct aspiration as a first pass technique (ADAPT) using a large-bore distal access catheter and primary aspiration for vessel recanalization. A recent publication indicated that the Solumbra technique may be the most efficient method for reducing the embolization of clot fragments at the time of mechanical thrombectomy while a BGC may be the best method for preventing clot fragmentation in the first place [7]. Thus, at our institution, we favor using the combination of Solumbra, enhanced with the aspiration retriever technique for stroke (ARTS), along with a BGC when anatomically feasible. With this thrombectomy setup, we have obtained great results in terms of recanalization and reperfusion rates, as well as a reduction in inadvertent embolization into distal/new territories [8]. We almost exclusively rely on this triaxial setup for anterior circulation AIS due to ELVO, with different permutations of the devices we use, e.g. 8 Fr. FlowGate balloon guide catheter (Stryker Neurovascular, Fremont, CA) vs. 9 Fr. Cello™ (eV3, Irvine, CA) as the BGC, 5MAX™ ACE (Penumbra, Alameda, CA) as the intracranial intermediate/aspiration catheter, and Trevo® Pro 14 vs. Trevo® Pro 18 microcatheters (Stryker Neurovascular, Fremont, CA) vs. Marksman™ microcatheter (Penumbra, Alameda, CA) vs. Excelsior^®^ XT-27^®^ (Stryker Neurovascular, Fremont, CA) as the microcatheter used for delivery of a Trevo® XP ProVue™ (Stryker Neurovascular, Fremont, CA) vs. Solitaire™ 2 stent-retriever device (eV3, Irvine, CA). Currently, the Penumbra aspiration catheters are the only FDA-approved devices in the US for the purposes of direct mechanical aspiration thrombectomy in the setting of AIS secondary to ELVO.

A new generation of intermediate/delivery catheters was recently introduced to the US market: the Arc™ catheter series (Medtronic, Irvine, CA), which includes the Arc™ and Arc™ mini intracranial support catheters. The Arc™ catheter is a flexible, single lumen, variable stiffness composite catheter with a straight tip configuration. It has a band-like radiopaque platinum marker at its distal tip, which allows for proper visualization under fluoroscopic guidance. The usable length of the Arc™ is 132 cm with a distal ID of 0.061”, allowing for improved trackability over guide wires or microcatheters in order to reach smaller vessels. The catheter has a distal OD (max.) of 0.071”. This smaller distal ID/OD ratio proved for improved distal flexibility. The catheter has a proximal ID of 0.069”, which again provides improved trackability when advancing the catheter over a guide wire or microcatheter. The proximal OD of the catheter (max.) is 0.0825”. The Arc™ catheter can be navigated over a guide wire with a minimum OD of 0.038”.

The Arc™ intracranial support catheter is used in a triaxial assembly, which is defined as a three catheter assembly, one inside the other, the so-called “tower of power”. The first catheter is the guide catheter, in the case of AIS, a BGC. The second catheter is the intermediate catheter, in this case, the Arc™. The delivery catheter is the third component of the triaxial assembly, and in the case of AIS, it corresponds to the microcatheter; it is called the delivery catheter as it is with this catheter that the occluded vessel is catheterized and then through it, the stent retriever is delivered.

In this paper, we report our initial technical experience with the Arc™ support catheter used as an intracranial intermediate/aspiration catheter (AC) as part of the Solumbra/ARTS, plus BGC triaxial assembly, in three different cases of AIS presenting with anterior circulation ELVO.

## Technical report

This technical report prospectively describes the procedural aspects and outcomes in three consecutive patients admitted to our institution and endovascularly treated for AIS due to ELVO of the anterior circulation.

The study was approved by the University of Massachusetts Institutional Review Board (#H00001860). The need for informed consent was waived.

In all three cases, a BGC was placed in the cervical ICA. Through the BGC, the Arc™ guide catheter (Medtronic, Irvine, CA) was advanced seamlessly over a microcatheter/microwire system up to the proximal aspect of the clot. The stent-retriever spanning the length of the occlusive clot was deployed using the push-and-fluff technique (PFT) [9]. The Arc™ catheter (AC) was then further navigated to engage the proximal aspect of the clot and was then connected to a Penumbra aspiration pump (Penumbra Inc., Alameda, CA). After the stent-retriever was allowed to intercalate with the clot for about four minutes, the BGC at the cervical ICA was inflated, and under constant aspiration, the stent-retriever was partially brought inside the AC following the aspiration retriever technique for stroke (ARTS) we previously described [8]. The results of this technique, in our experience, have proven to be effective in obtaining a higher rate of complete vessel recanalization and reperfusion, most often after the first pass, with a lower rate of distal embolization [8]. These improved outcomes are likely the synergistic and beneficial effect of the combination of this technique with PFT. The stent-retriever and AC were then both withdrawn from the patient and inspected for clot capture. Once these devices were removed, vigorous aspiration was applied to the BGC until the absence of debris (clot fragments) within the catheter lumen was confirmed. Then the balloon was deflated, and the guide catheter was reconnected to the heparinized saline flush. A follow-up head digital subtraction angiography (DSA) was performed to assess the outcomes of the thrombectomy. 

### Case 1

**Figure 1:**
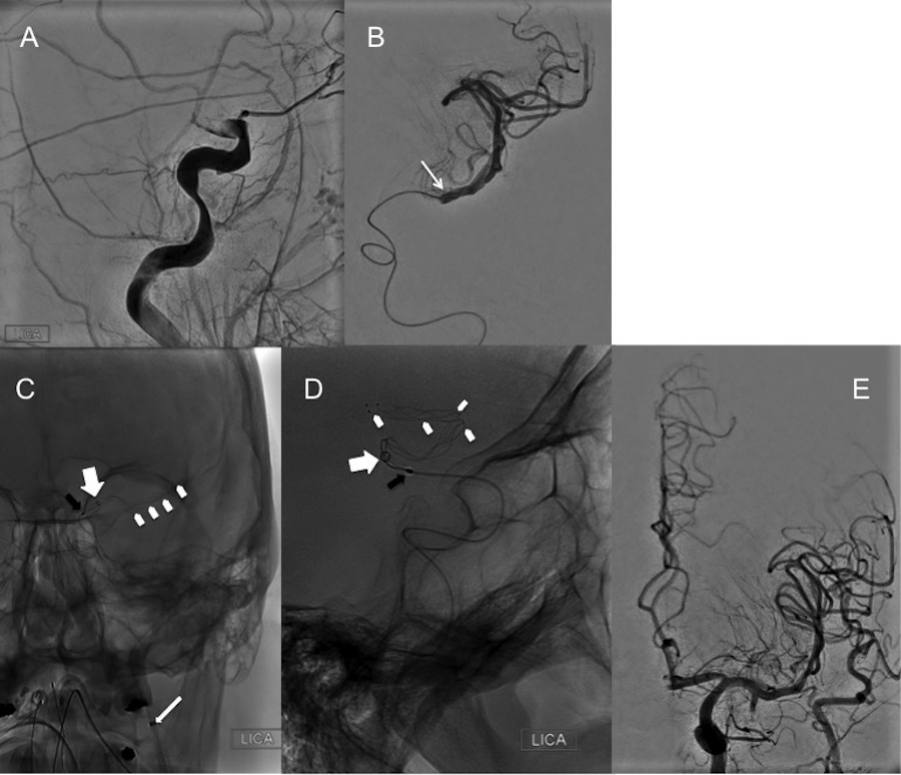
Case 1 (A) Magnified lateral oblique view of the high cervical and intracranial left ICA. There is no antegrade progression of contrast beyond the ophthalmic segment of the left ICA (white arrow). (B) Frontal DSA run through a microcatheter positioned distal to the clot in the left M1. (C) and (D) are frontal and lateral magnified radiographs moments prior to the thrombectomy; the BGC (long white arrow) has not yet been inflated and is distal to the recently placed cervical ICA stent, the tip of the Arc catheter is in the proximal left M1 (short white arrow), the microcatheter has been retracted following the PFT (tip is inside the Arc catheter, small black arrow), and the stent-retriever has been exposed (white arrowheads). (E) Final DSA run with a frontal view of the head demonstrates TICI 2B outcomes with residual occlusion of the left anterior temporal artery (long white arrow). LICA = Left internal carotid artery.

A 50-year-old male, modified Rankin scale (mRS) 0, presented with the acute onset of aphasia, right-sided facial droop, and right hemiplegia, with a National Institute of Health Stroke Scale (NIHSS) of 23. A computed tomography angiography (CTA) of the head and neck demonstrated a left cervical ICA dissection and an intracranial carotid “T” type of occlusion. A CT perfusion (CTP) indicated a large mismatch between the small core (infarct) and the penumbra (viable tissue at risk). The patient was not a candidate for the recombinant tissue plasminogen activator (rtPA), as the time of his stroke/symptom onset was unknown. He was thus referred to our service for endovascular treatment of his anterior circulation ELVO. The procedure was carried out under general anesthesia since the patient was agitated. In anticipation of the potential need for emergency extracranial carotid artery stenting, the patient was loaded with 600 mg of aspirin through an orogastric tube. Once the diagnostic angiogram had confirmed an occlusive dissection of the left cervical ICA, a Precise Pro RX 7x40 mm stent (Cordis, Miami, FL) was placed. Reestablishment of antegrade flow through the cervical left ICA then demonstrated an occlusion of the ipsilateral supraclinoid ICA just distal to the takeoff of the ophthalmic artery and extending up to the distal left M1 (Figures 1A-1B). As described in a recently proposed scheme to classify the tortuosity of the ICA, the cavernous carotid artery of this patient was a type IB (mild tortuosity) with open configuration/angles of the anterior and posterior genu, with the subcategory determined by the posterior genu angle, which, in his case, was equal to 90 degrees [10]. For this particular case, we used an 8 Fr FlowGate (Stryker Neurovascular, Fremont, CA) BGC positioned distal to the recently placed carotid stent, the Arc™ support catheter (Medtronic, Irvine, CA) as the intracranial intermediate AC, and a Marksman™ microcatheter (eV3, Irvine, CA), which was navigated over a Synchro-2 microwire (Stryker Neurovascular, Fremont, CA)  beyond the occluded left M1. For the first thrombectomy pass, a Trevo® XP ProVue 6 x 25 mm Retriever (Stryker Neurovascular, Fremont, CA) was deployed spanning from the distal left M1 segment down to the distal supraclinoid ICA. After the first thrombectomy pass, follow-up DSA runs demonstrated persistent occlusion of the most distal supraclinoid left ICA and ipsilateral M1 segment. A second thrombectomy pass was then performed, with the stent retriever spanning a proximal M2 branch down to the proximal M1 segment (Figures 1C-1D). Follow-up DSA runs showed recanalization of the left supraclinoid ICA and left M1. There was also complete patency of the left anterior cerebral artery (ACA). However, there was residual occlusion of the superior left M2 branch. A third thrombectomy pass was performed using a similar technique, this time with the Trevo® Pro 18 microcatheter (Stryker Neurovascular, Fremont, CA). The Trevo® Pro 18 microcatheter was then navigated slightly more distally, this time into the proximal superior division of the left M2 branch. A Trevo® XP ProVue 4 x 20 mm (Stryker Neurovascular, Fremont, CA) stent-retriever was then delivered across the occluded segment. Follow-up angiograms demonstrated complete patency of the left supraclinoid ICA, ACA, M1, and M2 branches. As the left M1 was recanalized, residual occlusion of the left anterior temporal artery was noted. Using a Trevo® XP ProVue 3 x 30 mm stent-retriever (Stryker Neurovascular, Fremont, CA), two additional thrombectomy passes of the left anterior temporal artery occlusion were performed. In each of these thrombectomy attempts, the Arc™ catheter was again navigated against the proximal aspect of the clot, this time, situated near the ostium of the left anterior temporal artery. It is unclear why recanalization of the left anterior temporal artery was unsuccessful. No further recanalization attempts were made as some retrograde filling of the vessel territory via leptomeningeal collaterals existed. Final DSA runs demonstrated thrombolysis in cerebral infarction (TICI) 2B flow through the left anterior cerebral circulation, with a cutoff of the left anterior temporal artery (Figure 1E). The procedure was then concluded, and the patient was extubated and transferred to the neurointensive care unit. By postoperative day (POD) 2, the patient had made significant improvement with an NIHSS of 8, with some mixed aphasia, and 4/5 strength in his right hemibody. At POD 30, the patient had only mild dysarthria and some word finding difficulty, near-normal motor strength, and had been discharged from physical therapy and occupational therapy but was still undergoing speech therapy.

### Case 2

**Figure 2:**
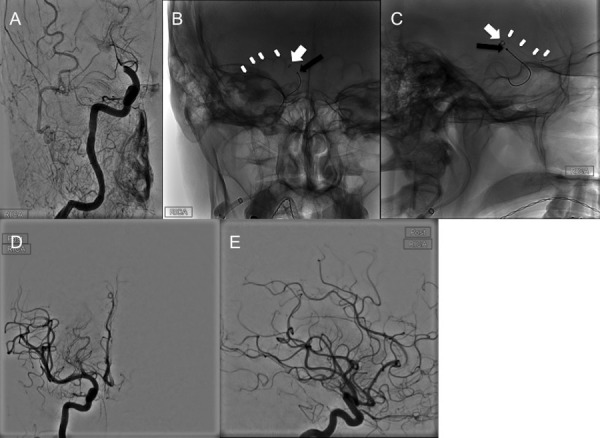
Case 2 (A) Frontal DSA run of the right anterior circulation demonstrates abrupt cutoff of the supraclinoid right ICA just distal to the right posterior communicating and right anterior choroidal arteries (long white arrow). The tip of the BGC is seen at the cervical ICA (short white arrow); this was soon repositioned slightly more proximal. (B) Frontal and (C) lateral magnified radiographs of the head demonstrating the triaxial assembly for mechanical thrombectomy for the second pass as follows: The inflated BGC (short black arrow) in the right cervical ICA, the distal marker of the intermediate Arc™ catheter facing the proximal aspect of the occluded right M1 segment (short white arrows), and the stent retriever which has been deployed from a proximal M2 branch down to the proximal M1 (white arrowheads). Note the microcatheter used to deliver the stent-retriever is inside the intermediate catheter (long black arrows). Prior to the thrombectomy pull the microcatheter was removed (ARTS technique). (D) and (E): Final DSA runs of the head with frontal and lateral views demonstrate a TICI 3 outcome. RICA = Right internal carotid artery.

A 59-year-old female with pre-stroke mRS 1 presented to the Emergency Department after an abrupt onset of left-sided hemiplegia and left hemineglect, with an NIHSS of 17. No rtPA was given as the time of stroke/symptom onset was unknown. CTA of the head and neck demonstrated occlusion of the right ICA terminus extending into the ipsilateral M1 and A1 segments (“T” occlusion). A hyperacute magnetic resonance image (MRI) of the brain demonstrated restricted diffusion within the right basal ganglia. Prior to any intervention, her stroke symptoms spontaneously improved to an NIHSS of 10 by the time the MRI was obtained. The patient was then referred to our service for mechanical thrombectomy. Since the patient was agitated, the procedure was carried out under general anesthesia. A DSA of the head confirmed the CTA findings (Figure 2A). The cavernous carotid artery of this patient was a type III (moderate), defined by the posterior deflection of the posterior genu, giving it a buckled appearance [10]. The triaxial assembly consisted of a 9 Fr Cello™ (ev3) for BGC, the Arc™ catheter (Medtronic) as the intracranial AC, and the Excelsior® XT-27® microcatheter (Stryker Neurovascular), which was, in turn, navigated over a Synchro-2® microwire (Stryker Neurovascular) into the right middle cerebral artery (MCA). The microcatheter was positioned just beyond the distal right M1 segment. A 6 x 20 mm Solitaire™ 2 stent-retriever (ev3) was deployed, spanning the distal right M1 down to the ipsilateral supraclinoid ICA. The Arc™ catheter was further navigated intracranially into the supraclinoid ICA at the proximal aspect of the clot (Figures 2B-2C). After the first thrombectomy pass, follow-up angiograms demonstrated recanalization of the supraclinoid ICA and right anterior cerebral artery (ACA) with residual occlusion of the right M1 segment. In a similar fashion, with the same triaxial assembly, the stent-retriever was deployed spanning the entire length of the right M1. The Arc™ catheter was again positioned against the proximal aspect of the clot. After the second thrombectomy pass, follow-up angiograms demonstrated complete recanalization of the right MCA and right ACA with a TICI 3 (Figures 2D-2E). The patient was transferred to the neurointensive care unit where, at POD 1, she had recovered to a 4/5 left hemibody strength with some mild dysarthria. By POD 3, the patient was transferred to a rehabilitation facility for further management with an NIHSS of 3.

### Case 3

**Figure 3:**
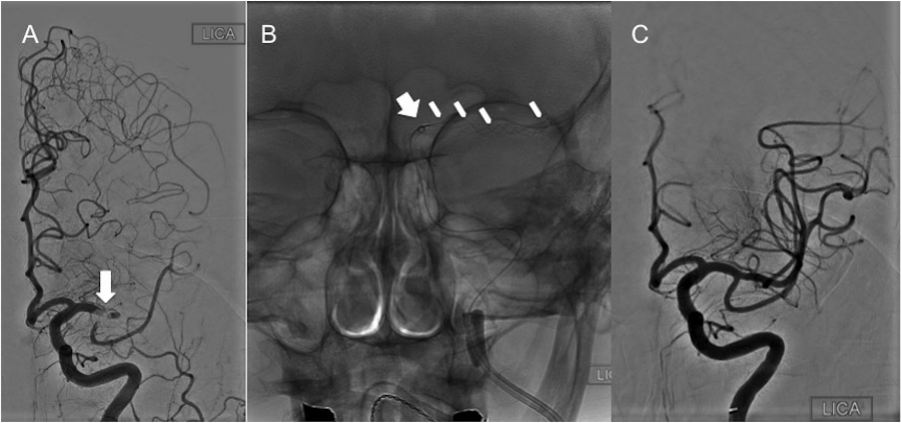
Case 3 (A) Frontal DSA run of the left anterior circulation demonstrating abrupt truncation of the left M1 segment (long white arrow). (B) The assembly for mechanical thrombectomy is seen as follows: The inflated BGC at the cervical ICA (short black arrow), distal marker of the intermediate Arc™ catheter facing the proximal aspect of the occluded left M1 segment (short white arrow), and the stent retriever which has been deployed from a proximal M2 branch down to the proximal M1 (white arrowheads). Note the microcatheter used to deliver the stent-retriever has been removed (ARTS technique). (C) Final frontal DSA run of the left anterior circulation demonstrating a TICI 2B angiographic outcome. LICA = Left internal carotid artery.

A 91-year-old female, pre-stroke mRS of 1, presented with the acute onset of aphasia, right facial droop, and right hemiparesis, with an NIHSS of 23. She was a “wake-up” stroke and, thus, not a candidate for rtPA. A non-contrast CT of the head demonstrated an Alberta Stroke Program Early CT Score (ASPECTS) of 9 with subtle hypodensity in the left insular ribbon. A CTA of the head and neck demonstrated occlusion of the left M1 segment with limited collateral circulation. She was taken to the angiography suite for a thrombectomy under general anesthesia, as she was confused and agitated. The triaxial assembly included an 8 Fr FlowGate (Stryker Neurovascular) for BGC at the cervical left ICA, the Arc™ catheter (Medtronic) used as the intermediate AC, and a Trevo® Pro 18 microcatheter over a Synchro-2® microwire (Stryker Neurovascular). The cavernous carotid artery of this patient was a type IB (mild tortuosity) with open configuration/angles of the anterior and posterior genu, with the subcategory determined by the posterior genu angle, which, in her case, was equal to 90 degrees [10]. The horizontal segment of this patient’s cavernous carotid lacked any tortuosity. Angiographic runs determined that the clot was extending from the mid-left M1 to the left MCA bifurcation (Figure 3A). After traversing the clot with the microcatheter/microwire system, a Trevo® XP ProVue 4 x 20 mm stent-retriever (Stryker Neurovascular) was deployed (Figure 3B). With only a single thrombectomy pass, there was complete recanalization of the left MCA with a TICI 3 angiographic outcome (Figure 3C). The patient was extubated, and at about one hour after the procedure, her right upper mobility and strength were significantly improved. By POD 4, her NIHSS was down to 3, with some subtle dysarthria, right facial droop, and 4/5 strength in her right upper extremity.

## Discussion

The aim of this technical report is to describe our initial experience with the newest generation of intracranial support catheters, the Arc™ Support Catheter, used as an intracranial intermediate aspiration catheter in a triaxial assembly as detailed above for treatment of anterior circulation AIS due to ELVO. Patient demographics, characteristics of vessel occlusion, permutations of the triaxial assemblies used, and final TICI scores, as well as clinical outcomes, are outlined in Table 1. It is important to highlight that none of the three patients presented here had any significant tortuosity in the cervical ICA. There were no areas of elongation, redundancy, or “S” configurations and also no evidence of abrupt angulation of the vessel axis in any of the patients [11-12]. Hence, the positioning of the BGC at the cervical ICA and subsequent navigation of the Arc™ catheter, as part of a triaxial assembly, proved quite uneventful. In Case 1 (tandem lesions), there were also no difficulties when crossing the recently stented proximal ICA.

**Table 1:** Patient Demographics, Characteristic of Vessel Occlusion, Thrombectomy Assembly Used, and Outcomes

						Assembly Used for Thrombectomy							
Case	Gender	Age (Years)	Occluded Site	Pre-stroke mRS	NIHSS	Balloon Guide Catheter	Intermediate (Aspiration) Catheter	Micro-catheter(s)	Stent-retriever	Number of Thrombectomy Passes	Time From Groin Puncture to Recanalization (Min)	TICI	mRS at 30 Days
1	Male	50	Tandem lesions, left cervical ICA and left carotid "T" occlusion	0	23	8 Fr FlowGate	Arc™ Catheter	Marksman™, Trevo^®^ Pro 18	Trevo^® ^XP ProVue 4 x 20 mm; Trevo® XP ProVue 3 x 20 mm	3	106	2B	0
2	Female	59	Right carotid “T’ occlusion	1	10	9 Fr Cello	Arc™ Catheter	Excelsior XT-27	Solitaire™ 2 6 x 30 mm; Solitaire 2 6 x 20 mm	2	65	3	1
3	Female	91	Left M1 to left MCA bifurcation occlusion	1	23	8 Fr FlowGate	Arc™ Catheter	Trevo^®^ Pro 18	Trevo^® ^XP ProVue 4 x 20 mm	1	23	3	1

**Figure 4:**
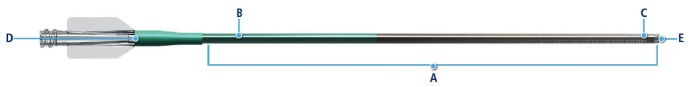
The Arc™ catheter

In all of these cases, the Arc™ catheter, as part of a triaxial assembly, was easily navigated through the cervical and intracranial ICAs. All three patients did well after their procedures with no associated periprocedural complications, such as symptomatic intracranial hemorrhage or worsening of their stroke symptoms. They were all eventually discharged to rehabilitation facilities with an overall positive prognosis from their neurological standpoint. Figure 4 shows the Arc™ catheter, and the accompanying Table 2 details the different specification of the device (e.g., working length, proximal outer diameter, distal outer diameter, etc.). In the United States, the Arc™ Intracranial Support Catheter is indicated for the introduction of interventional devices into the peripheral and neurovasculature. In our case series, this catheter was not only used for this purpose but also for aspiration (off-label use), while the stent-retriever was intercalating with the clot and then during extraction of the stent-retriever/clot under constant aspiration. There were no complications with the use of the Arc™ catheter.

**Table 2:** Specifications of the Arc™ Catheter

Name	Working Length (A)	Proximal Outer Diameter (B)	Distal Outer Diameter (C)	Proximal Inner Diameter (D)	Distal Inner Diameter (E)	Max Wire Compability
Arc™	132 cm	0.080” / 6.1F	0.069” / 5.3F	0.069” / 5.3F	0.061” / 4.7F	0.038”

## Conclusions

As an initial and limited report, the Arc™ catheter was shown to be secure and easy to navigate as part of a triaxial system used to treat anterior circulation AIS due to ELVO, with excellent trackability, pushability, flexibility, and conformability, while still providing adequate support for both the delivery microcatheter and subsequent stent-retriever device. Overall, in these three cases, two different BGCs (Cello™ and FlowGate), three different microcatheters (Marksman™, Trevo^®^ Pro 18, and Excelsior^®^ XT-27^®^), and two different families of stent-retrievers (Trevo^®^ XP ProVue and Solitaire™ 2) were used. There were no complications related to the usage of any of the devices, including the Arc™ catheter. The Arc™ support catheter may be used as an intermediate aspiration catheter for treating ELVO in AIS cases. Additional testing of this catheter in the setting of AIS and ELVO is required, particularly when treating AIS patients with a challenging and more tortuous anatomy of both their cervical or intracranial ICA segments.

## References

[1] Powers WJ, Derdeyn CP, Biller J, Coffey CS, Hoh BL, Jauch EC, Johnston KC, Johnston SC, Khalessi AA, Kidwell CS, Meschia JF, Ovbiagele B, Yavagal DR; American Heart Association Stroke Council (2015). 2015 American Heart Association/American Stroke Association Focused Update of the 2013 Guidelines for the Early Management of Patients With Acute Ischemic Stroke Regarding Endovascular Treatment: A Guideline for Healthcare Professionals From the American Heart Association/American Stroke Association. Stroke.

[2] Berkhemer OA, Fransen PS, Beumer D, van den Berg LA, Lingsma HF, Yoo AJ, Schonewille WJ, Vos JA, Nederkoorn PJ, Wermer MJ, van Walderveen MA, Staals J, Hofmeijer J, van Oostayen JA, Lycklama à Nijeholt GJ, Boiten J, Brouwer PA, Emmer BJ, de Bruijn SF, van Dijk LC, Kappelle LJ, Lo RH, van Dijk EJ, de Vries J, de Kort PL, van Rooij WJ, van den Berg JS, van Hasselt BA, Aerden LA, Dallinga RJ, Visser MC, Bot JC, Vroomen PC, Eshghi O, Schreuder TH, Heijboer RJ, Keizer K, Tielbeek AV, den Hertog HM, Gerrits DG, van den Berg-Vos RM, Karas GB, Steyerberg EW, Flach HZ, Marquering HA, Sprengers ME, Jenniskens SF, Beenen LF, van den Berg R, Koudstaal PJ, van Zwam WH, Roos YB, van der Lugt A, van Oostenbrugge RJ, Majoie CB, Dippel DW; MR CLEAN Investigators (2015). A randomized trial of intraarterial treatment for acute ischemic stroke. N Engl J Med.

[3] Campbell BC, Mitchell PJ, Kleinig TJ, Dewey HM, Churilov L, Yassi N, Yan B, Dowling RJ, Parsons MW, Oxley TJ, Wu TY, Brooks M, Simpson MA, Miteff F, Levi CR, Krause M, Harrington TJ, Faulder KC, Steinfort BS, Priglinger M, Ang T, Scroop R, Barber PA, McGuinness B, Wijeratne T, Phan TG, Chong W, Chandra RV, Bladin CF, Badve M, Rice H, de Villiers L, Ma H, Desmond PM, Donnan GA, Davis SM; EXTEND-IA Investigators (2015). Endovascular therapy for ischemic stroke with perfusion-imaging selection. N Engl J Med.

[4] Goyal M, Demchuk AM, Menon BK, Eesa M, Rempel JL, Thornton J, Roy D, Jovin TG, Willinsky RA, Sapkota BL, Dowlatshahi D, Frei DF, Kamal NR, Montanera WJ, Poppe AY, Ryckborst KJ, Silver FL, Shuaib A, Tampieri D, Williams D, Bang OY, Baxter BW, Burns PA, Choe H, Heo JH, Holmstedt CA, Jankowitz B, Kelly M, Linares G, Mandzia JL, Shankar J, Sohn SI, Swartz RH, Barber PA, Coutts SB, Smith EE, Morrish WF, Weill A, Subramaniam S, Mitha AP, Wong JH, Lowerison MW, Sajobi TT, Hill MD; ESCAPE Trial Investigators (2015). Randomized assessment of rapid endovascular treatment of ischemic stroke. N Engl J Med.

[5] Saver JL, Goyal M, Bonafe A, Diener HC, Levy EI, Pereira VM, Albers GW, Cognard C, Cohen DJ, Hacke W, Jansen O, Jovin TG, Mattle HP, Nogueira RG, Siddiqui AH, Yavagal DR, Baxter BW, Devlin TG, Lopes DK, Reddy VK, du Mesnil de Rochemont R, Singer OC, Jahan R; SWIFT PRIME Investigators (2015). Stent-retriever thrombectomy after intravenous t-PA vs. t-PA alone in stroke. N Engl J Med.

[6] Jovin TG, Chamorro A, Cobo E, de Miquel MA, Molina CA, Rovira A, San Román L, Serena J, Abilleira S, Ribó M, Millán M, Urra X, Cardona P, López-Cancio E, Tomasello A, Castaño C, Blasco J, Aja L, Dorado L, Quesada H, Rubiera M, Hernandez-Pérez M, Goyal M, Demchuk AM, von Kummer R, Gallofré M, Dávalos A; REVASCAT Trial Investigators (2015). Thrombectomy within 8 hours after symptom onset in ischemic stroke. N Engl J Med.

[7] Chueh JY, Puri AS, Wakhloo AK, Gounis MJ (2016). Risk of distal embolization with stent retriever thrombectomy and ADAPT. J Neurointerv Surg.

[8] Massari F, Henninger N, Lozano JD, Patel A, Kuhn AL, Howk M, Perras M, Brooks C, Gounis MJ, Kan P, Wakhloo AK, Puri AS (2016). ARTS (Aspiration-Retriever Technique for Stroke): Initial clinical experience. Interv Neuroradiol.

[9] Haussen DC, Rebello LC, Nogueira RG (2015). Optimizing clot retrieval in acute stroke: The push and fluff technique for closed-cell stentrievers. Stroke.

[10] Lin LM, Colby GP, Jiang B, Uwandu C, Huang J, Tamargo RJ, Coon AL (2015). Classification of cavernous internal carotid artery tortuosity: a predictor of procedural complexity in Pipeline embolization. J Neurointerv Surg.

[11] Metz H, Murray-Leslie RM, Bannister RG, Bull JW, Marshall J (2016). Kinking of the internal carotid artery. Lancet.

[12] Zenteno M, Leeb A, Moscote-Salazar LR (2016). Anatomic variations of the internal carotid artery: implications for the neurologic endovascular therapist (Article in Spanish). Bol Asoc Med P R.

